# Altered microbiome of serum exosomes in patients with acute and chronic cholecystitis

**DOI:** 10.1186/s12866-024-03269-6

**Published:** 2024-04-20

**Authors:** Qing Zhu, Min-Xian Li, Ming-Chin Yu, Qi-Wen Ma, Ming-Jie Huang, Chun-Wei Lu, Chun-Bing Chen, Wen-Hung Chung, Chih-Jung Chang

**Affiliations:** 1 Department of Surgery, Xiamen Chang Gung Hospital Hua Qiao University, Fujian, China; 2 School of Medicine and Medical Research Center, Xiamen Chang Gung Hospital Hua Qiao University, Fujian, China; 3 Drug Hypersensitivity Clinical and Research Center, Department of Dermatology, Chang Gung Memorial Hospital, Taoyuan, Linkou Taiwan; 4 College of Medicine, Chang Gung University, Taoyuan, Taiwan; 5 Department of Dermatology, Xiamen Chang Gung Hospital, Fujian, China

**Keywords:** Cholecystitis, Exosomes, Microbiota composition, 16S rRNA sequencing

## Abstract

**Background:**

This study aimed to investigate the differences in the microbiota composition of serum exosomes from patients with acute and chronic cholecystitis.

**Method:**

Exosomes were isolated from the serum of cholecystitis patients through centrifugation and identified and characterized using transmission electron microscopy and nano-flow cytometry. Microbiota analysis was performed using 16S rRNA sequencing.

**Results:**

Compared to patients with chronic cholecystitis, those with acute cholecystitis exhibited lower richness and diversity. Beta diversity analysis revealed significant differences in the microbiota composition between patients with acute and chronic cholecystitis. The relative abundance of *Proteobacteria* was significantly higher in exosomes from patients with acute cholecystitis, whereas *Actinobacteria*, *Bacteroidetes*, and *Firmicutes* were significantly more abundant in exosomes from patients with chronic cholecystitis. Furthermore, functional predictions of microbial communities using Tax4Fun analysis revealed significant differences in metabolic pathways such as amino acid metabolism, carbohydrate metabolism, and membrane transport between the two patient groups.

**Conclusions:**

This study confirmed the differences in the microbiota composition within serum exosomes of patients with acute and chronic cholecystitis. Serum exosomes could serve as diagnostic indicators for distinguishing acute and chronic cholecystitis.

**Supplementary Information:**

The online version contains supplementary material available at 10.1186/s12866-024-03269-6.

## Introduction

Cholecystitis, caused by cholecystolithiasis, microbial infection, ischemia, allergic reaction, and chemical damage, is one of the common digestive tract diseases [1]. Based on the duration of the condition, cholecystitis can be classified into acute and chronic types [2]. Acute cholecystitis is often triggered by gallstone obstruction or other lesions causing biliary obstruction, leading to bile stasis and gallbladder inflammation. It is a common cause of acute abdominal pain [3]. According to statistical data, the incidence of acute cholecystitis is approximately 7.45-10.06%, with a higher prevalence among the elderly, reaching 5-10% [4]. Although most cases of acute cholecystitis can be cured through conservative treatment or open surgery, improper management may result in severe complications such as pancreatitis, cirrhosis, liver failure, and even life-threatening situations [5–7]. Chronic cholecystitis, on the other hand, is a prevalent chronic gastrointestinal disorder, accounting for 60-80% of all cholecystitis cases. Its main symptoms include periodic or persistent mild to moderate pain [8–10]. While the prognosis for chronic cholecystitis is generally favorable, prolonged inflammation and damage may increase the risk of developing gallbladder cancer [11]. Currently, the treatment strategies differ between acute and chronic cholecystitis. Acute cases necessitate timely cholecystectomy or percutaneous cholecystostomy for high-risk patients, coupled with antibiotic therapy. Conversely, chronic cholecystitis can often be treated through elective cholecystectomy [12]. Therefore, it is crucial to diagnose and differentiate between acute and chronic cholecystitis accurately and promptly. This differentiation is vital in avoiding potential complications associated with emergency surgery or intervention, as well as preventing disease progression into complicated gallbladder conditions.

The human microbiome refers to the diverse array of bacteria, viruses, fungi, protozoa, and archaea present on and inside the human body [13]. Scientific evidence indicates that only 1% of human DNA originates from the human genome, while the remaining 99% is derived from the microbiome [14]. Although most microbes may not directly harm the human body, they exert a significant influence on human health and are closely associated with the onset and progression of various diseases [15]. Within the human gallbladder and bile, a diverse microbial community exists. Several studies have identified different bacterial communities from bile and gallbladder walls of cholecystitis patients through microbial isolation and culture methods [16]. Research has demonstrated that dysbiosis in the microbiome plays a crucial role in the development of cholecystitis [17]. However, the differences in microbial composition between patients with acute and chronic cholecystitis remain unclear. Therefore, investigating the microbial composition of patients with acute and chronic cholecystitis is essential for the diagnosis of these conditions.

Exosomes are small vesicles with a diameter of approximately 30–150 nm, released by cells and found in various bodily fluids [18]. Exosomes function by encapsulating diverse genetic materials, including DNA fragments, and RNA, as well as functional proteins such as enzymes and receptors. They transport these materials to target cells, regulating gene expression, cell signaling, and other biological processes within the target cells [19]. During cholecystitis, exosomes release inflammation mediators and regulatory factors, leading to sustained or exacerbated inflammatory responses [20]. Additionally, the genetic materials within exosomes may participate in regulating gene expression and signaling pathways associated with cholecystitis, further influencing the development and progression of the condition [21]. Studies have indicated that certain bacteria can directly enter exosomes, inducing exosome release and affecting the quality and composition of exosomes [22]. Exosomes derived from infected cells or those carrying microorganisms such as bacteria and viruses play a significant role in host immune responses and disease progression [23]. Hence, investigating the microbial composition within exosomes from patients with acute and chronic cholecystitis is essential for gaining a deeper understanding of the underlying mechanisms and diagnostic and therapeutic approaches for these conditions. However, there is no reported evidence regarding whether the differences in microbial composition carried by exosomes in patients with acute and chronic cholecystitis impact the development of the condition.

In this study, we utilized 16 S rRNA sequencing technology to analyze the microbial composition within serum exosomes of patients with acute and chronic cholecystitis. Simultaneously, we identified potential metabolic pathways within the detected microbial communities. Our research delineated the microbial distinctions in serum exosomes between acute and chronic cholecystitis, providing novel insights into the pathogenesis and diagnosis of these conditions.

## Materials and methods

### Sample collection

A total of 39 serum samples were collected from 17 patients with acute cholecystitis and 22 patients with chronic cholecystitis. Patients clinically diagnosed with acute cholecystitis were categorized as the Acute group (*n* = 17), while those diagnosed with chronic cholecystitis were classified as the Chronic group (*n* = 22). No participants had taken probiotics, antibiotics, or related medications within 60 days before the study. All participants were free from severe organic diseases such as heart, lung, kidney, and other infectious diseases. Written informed consent was obtained from all participants. The research protocol was approved by the Ethics Committee of Xiamen Chang Gung Hospital (NO. CMRPG1G0161).

### Isolation of exosomes

The patients’ serum specimens were subjected to a 300 g low-speed centrifugation for 10 min to remove cellular components. Subsequently, a 2000 g low-speed centrifugation for 10 min was performed to eliminate dead cells, followed by a 10,000 g centrifugation for 70 min to eliminate cell debris. Next, a 120,000 g high-speed centrifugation for 60 min was conducted to obtain pelletized extracellular vesicles. The vesicles were resuspended in an appropriate volume of PBS to eliminate contaminating proteins and subjected to another 120,000 g centrifugation for 60 min. The precipitate was collected and resuspended in PBS, and the samples were stored at -80 °C.

### Characterization of exosomes

The morphology of exosomes was examined using transmission electron microscopy (TEM) with negative staining. The isolated exosomes were stained with 2% uranyl acetate for 1 min in a total volume of 10 mL, placed onto grids, and observed using a TEM operating at 80 kV (JEM-1400Plus, JEOL Ltd, Tokyo, Japan). Additionally, following previously reported experimental methods from our laboratory [24, 25], exosomes concentration and size in serum were analyzed using nano-flow cytometry (nFCM) with a nanoanalyzer, following the manufacturer’s instructions.

### DNA extraction from exosomes

The exosomes were lysed using an appropriate amount of pancreatic protease to release internal DNA. Following the manufacturer’s instructions precisely, total DNA was extracted from the lysate of extracellular vesicles using a DNA extraction kit (Rengenbio, China). The concentration of DNA was determined using the NanoDrop assay, and the integrity and purity of DNA were evaluated through 1% agarose gel electrophoresis.

### 16S amplification and deep sequencing

To characterize the microbiota within extracellular vesicles, we employed a novel method for amplification and sequencing of 16S rRNA. This method involves amplification of 68% of the bacterial 16S rRNA gene using short amplicons [26]. Previous studies have provided comprehensive details regarding this methodology [27]. The amplification of five regions of the 16S rRNA gene was performed using 100 ng of DNA, a set of 10 multiplex primers, 0.2 mM dNTPs sourced from Larova GmbH, and 0.02 U/µL of Phusion Hot Start II DNA Polymerase (Thermo Scientific). After combining 40–50 amplicons into sub-libraries, each sub-library underwent purification utilizing the Qiaquick PCR purification kit (QIAGEN, Germany) as per the manufacturer’s guidelines. Subsequently, multiple sub-libraries were pooled to create the final library. Subsequently, further purification from primer dimers was conducted using Agencourt AMPure XP (Beckman Coulter) at a volumetric ratio of 1:0.85 (Library: beads), followed by supplementation of the library with 15% PhiX (8 pM). Finally, paired-end sequencing was performed on the Illumina NovaSeq 6000 platform, generating paired-end reads with a length of 150 bp.

### Microbiome analyses

After sequencing data were obtained, demultiplexing was performed for each sample based on barcode recognition. Subsequently, the cutadapt plugin was utilized to remove additional primers from the paired-end reads. The sequences were then subjected to quality filtering, denoising, merging, and chimera removal using the DADA2 plugin [28]. To explore the taxonomic composition of each sample, operational taxonomic units (OTUs) were clustered at 97% sequence similarity. Venn diagrams were generated to compare the shared OTUs between the two sample groups. Taxonomic annotation was carried out using the Greengenes_12_8 database with QIIME2 software, providing relative abundance information for each sample [29]. For analyzing the microbial communities in exosomes between the two sample groups, α-diversity and β-diversity analyses were conducted. Alpha-diversity analysis reflects the complexity and diversity of species within each sample and was computed using QIIME2 software, including observed species, ACE, Simpson, and Shannon indices. Weighted and unweighted UniFrac distances were calculated using QIIME 2 software. Principal coordinates analysis (PCoA) was visualized using ggplot2 and ade packages in R, and arithmetic distances were interpreted using an unweighted pair-group method with arithmetic mean (UPGMA) hierarchical clustering based on the average linkage algorithm for clustering analysis.

Furthermore, to analyze the compositional changes of microbial communities within exosomes between the two sample groups, Metastat software was employed for relative abundance analysis of microbial communities at the phylum, class, order, family, and genus levels. Linear discriminant analysis effect size (LEfSe) was utilized to assess the effect size of significantly different taxa at different taxonomic levels. Tax4Fun package in R software was used for functional prediction of microbial communities within extracellular vesicles based on the 16S SILVA database [30]. 16S rRNA gene sequences were extracted from the KEGG prokaryotic genome database [31], and aligned to the SILVA SSU Ref NR database using the BLASTN algorithm to create a related matrix [32]. The annotated functional information of the KEGG prokaryotic genome was mapped to the SILVA database using UproC and PAUDA methods for functional annotation. Finally, OTUs were clustered from the sequenced samples using SILVA database sequences as reference sequences to obtain functional annotation information.

### Statistical analysis

All experimental data were statistically analyzed using GraphPad Prism version 8.0 (GraphPad Software, San Diego, CA, USA) and presented as mean ± standard deviation (SD). Differences between the two groups in extracellular vesicle size and α-diversity analysis were evaluated using unpaired t-tests, while the significance of other differences in distributions was assessed using Kruskal-Wallis tests. A *P*-value < 0.05 was considered statistically significant.

## Results

### Clinical characteristics of participants

The demographic information of all participants is presented in Table 1. The age range of the participants varied from 26 to 71 years old. In the Acute group, the average age of patients was (51 ± 11) years, with a white blood cell (WBC) count of (8.83 ± 3.43)×10^9^/L and C-reactive protein (CRP) levels of (24.02 ± 41.45) mg/L. In the Chronic group, the average age of patients was (45 ± 12) years, with a WBC count of (6.65 ± 2.06)×10^9^/L and CPR levels of (1.21 ± 0.88) mg/L.

**Table 1:** Characteristics of patients with acute and chronic cholecystitis

Variables	Acute group	Chronic group	*P*-value
Subjects (N)	17	22	-
Gender (M/F)	7/10	4/18	-
Age (years)	51 ± 11	45 ± 12	< 0.05
WBC(10^9^/L)	8.83 ± 3.43	6.65 ± 2.06	< 0.05
CRP(mg/L)	24.02 ± 41.45	1.21 ± 0.88	< 0.05

*WBC* White blood cell, *CRP* C-reactive protein, *M* Male, *F* Female

### Phenotypic characterization of exosomes

**Fig. 1:**
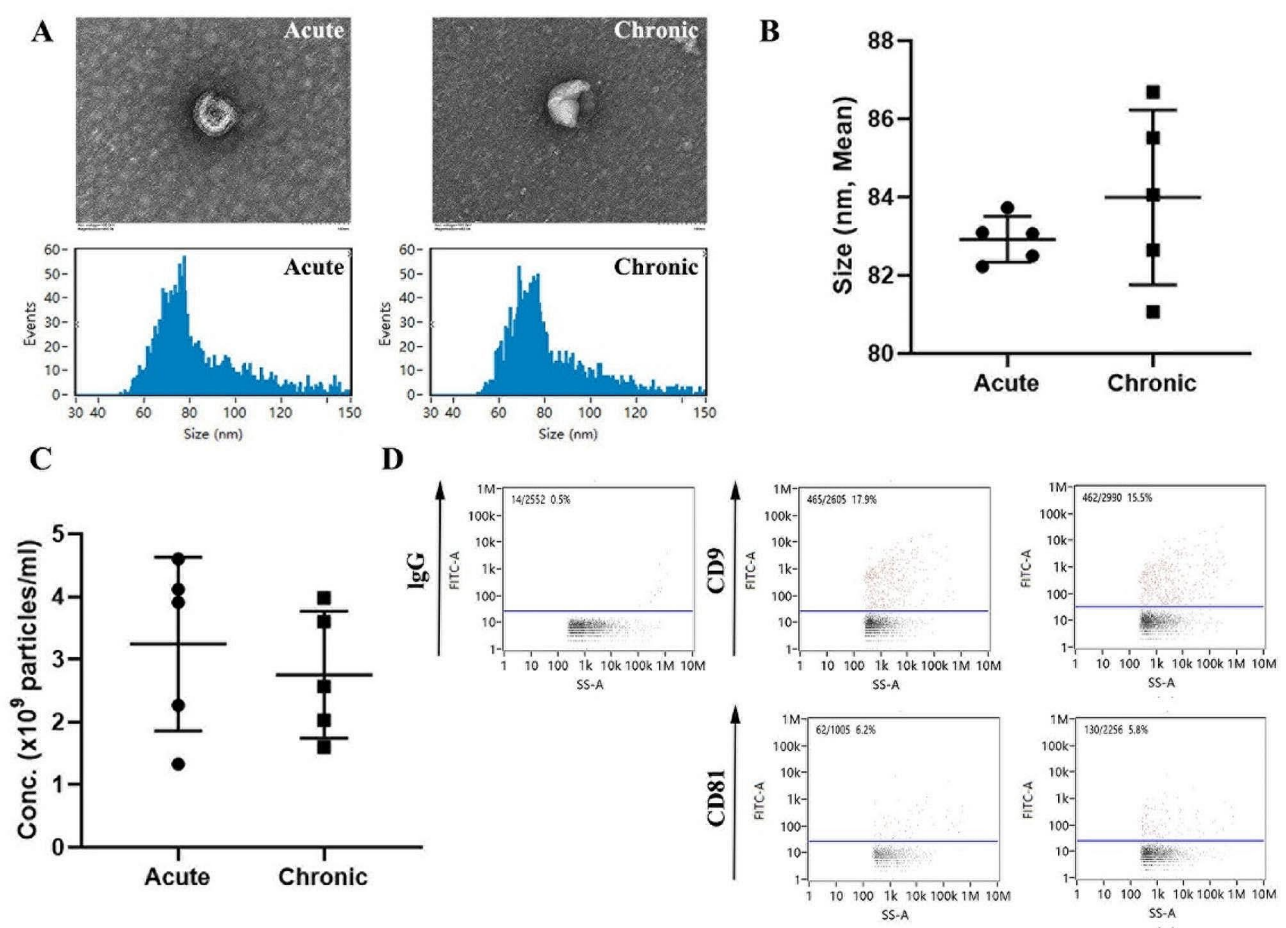
The characterization of exosomes from the Acute group and Chronic group. **A** Observation of electron micrographs of exosomes by transmission electron microscopy (TEM) (Bar = 100 nm). **B** Measurement of exosomes diameter using nanoflow cytometry (nFCM). **C** Measurement of exosomes concentration using nFCM. **D** Detection of surface markers CD9 and CD81 expression on exosomes using nFCM, with blank IgG used as a negative control.

Exosomes were isolated from both the Acute and Chronic groups and identified and confirmed using TEM. TEM analysis revealed that the exosomes exhibited typical cup-shaped vesicles (Fig. 1A). There were no significant differences in the average diameter and concentration of exosomes between the Acute and Chronic groups (Fig. 1B, C). Purity analysis of exosomes was conducted using nFCM, which showed positive surface markers (CD9 and CD81) compared to the blank IgG control group (Fig. 1D). These results demonstrate the successful isolation of exosomes in this study. Overall, these findings indicate the effective separation of exosomes.

### Difference of microbiota between acute group and chronic group

**Fig. 2:**
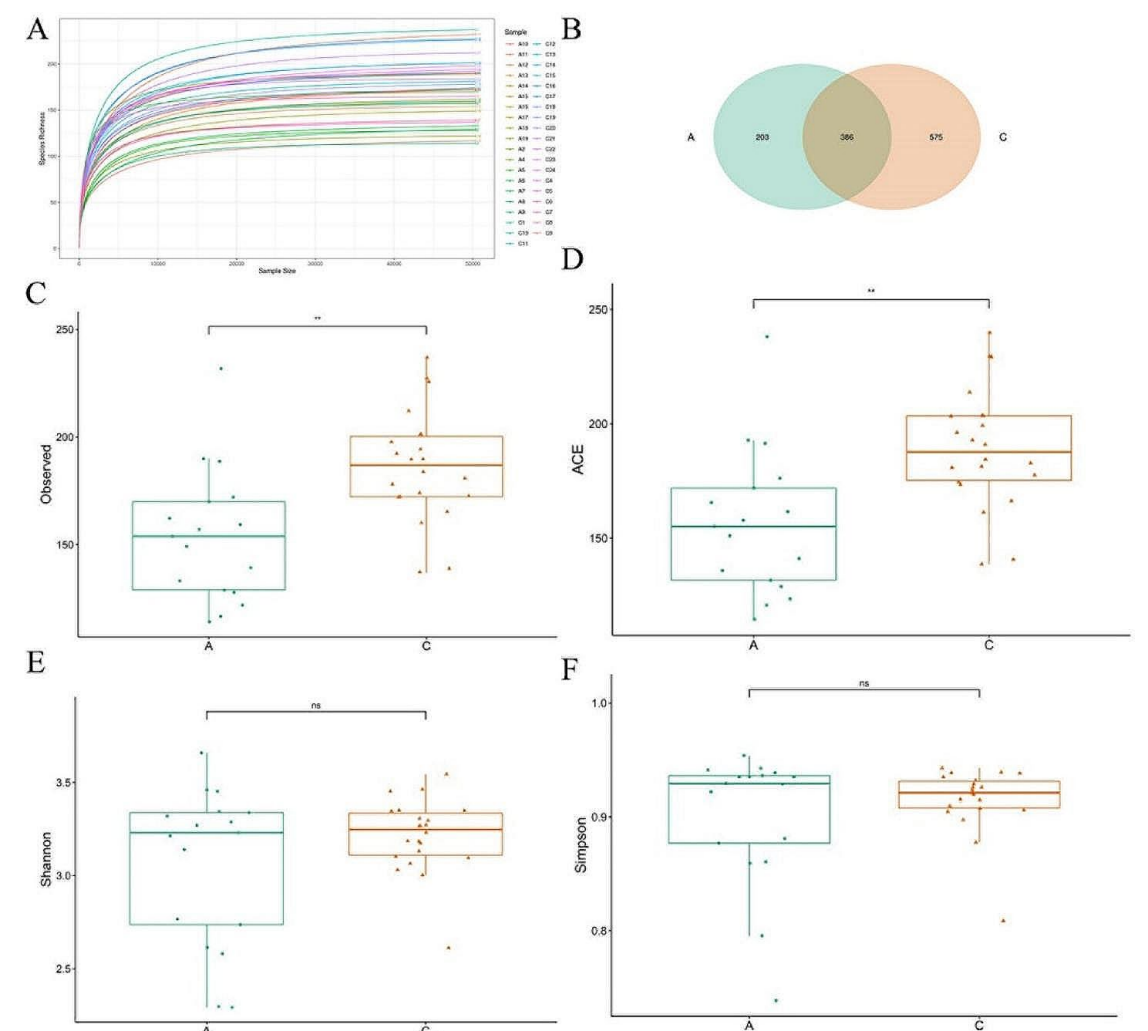
Alpha diversity analysis of microbial communities in exosomes of patients with acute and chronic cholecystitis. **A** The rarefaction curve. **B** Venn diagram showing the distribution of OTUs in the Acute and Chronic groups. **C** Box plot depicting the number of observed species (Observed_species index). **D** Abundance-based Coverage Estimator (ACE) used to estimate the number of unobserved species and calculate the estimation of the total species count. **E** Shannon index reflecting species richness and evenness. **F** Simpson index measuring species diversity and evenness within the community. ^**^P < 0.01. ns, no significant difference. A: Acute group; B: Chronic group

**Fig. 3:**
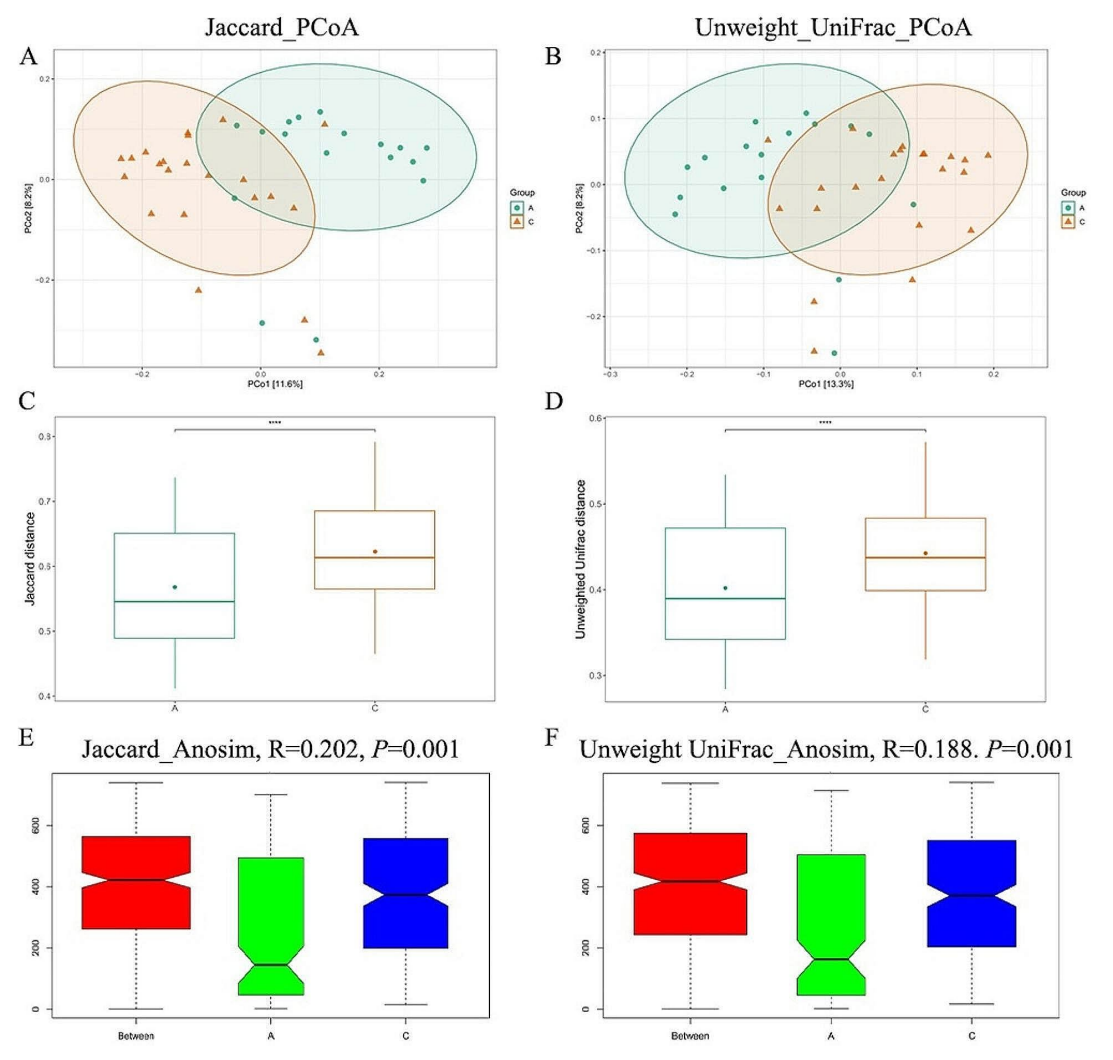
Beta diversity analysis of microbial communities in exosomes of patients with acute and chronic cholecystitis. **A** Investigation of β-diversity changes in microbial communities within exosomes using Jaccard_PcoA analysis between the two groups. **B** Investigation of β-diversity changes in microbial communities within exosomes using Unweighted UniFrac_PcoA analysis between the two groups. **C** Jaccard distances between samples from the two groups. **D** Unweighted_UniFrac distances between samples from the two groups. **E** Anosim analysis of Jaccard dissimilarities. **F** Anosim analysis of Unweighted_UniFrac dissimilarities. ^***^P < 0.001

The rarefaction curve analysis revealed a sharp increase followed by a plateau for each sample, indicating that the sequencing depth was sufficient to reflect the microbial diversity in exosomes (Fig. 2A). The sequences were clustered and classified into operational taxonomic units (OTUs) with 97% similarity, resulting in a total of 1164 OTUs across both groups (Supplementary Table S1). The Venn analysis showed 589 OTUs for the Acute group and 961 OTUs for the Chronic group, with 386 OTUs shared between the two groups (Fig. 2B), indicating both similarities and differences in microbial communities within exosomes of patients from these two groups. To further analyze the changes in microbial communities within exosomes of the two groups, this study conducted α-diversity analysis, including the Observed species index representing richness, Shannon and Simpson indices representing diversity, and Abundance-based Coverage Estimator (ACE) evaluating abundance coverage. The α-diversity analysis results demonstrated that, compared to the Chronic group, the Acute group exhibited decreased Observed_species index (*P* < 0.01) and ACE index (*P* < 0.01) as well as reduced Shannon index (Fig. 2C–E). Conversely, the Simpson index was higher (Fig. 2F), indicating a higher abundance and diversity of microbial communities in the exosomes of the Chronic group. Furthermore, β-diversity analysis was conducted to estimate the similarity of microbial communities between the two groups. Distances between samples were calculated using Jaccard and Unweighted_UniFrac metrics, and Principal Coordinates Analysis (PCoA) was performed on the distance matrices. Jaccard_PcoA and Unweighted_UniFrac_PcoA analysis results showed significant separation between the two groups (Fig. 3A, B), and both Jaccard and Unweighted_UniFrac distances exhibited significant differences between the two groups (*P* < 0.001) (Fig. 3C, D). Additionally, Anosim analysis was employed to assess the significance of β-diversity, revealing statistically significant differences in microbial community composition between the two groups (Jaccard_Anosim: 0.202, *P* = 0.001; Unweighted_UniFrac_Anosim: *R* = 0.188, *P* = 0.001). These results indicated significant differences in microbial communities within the exosomes of patients from the two groups.

### Bacterial composition at different taxonomic levels

**Fig. 4:**
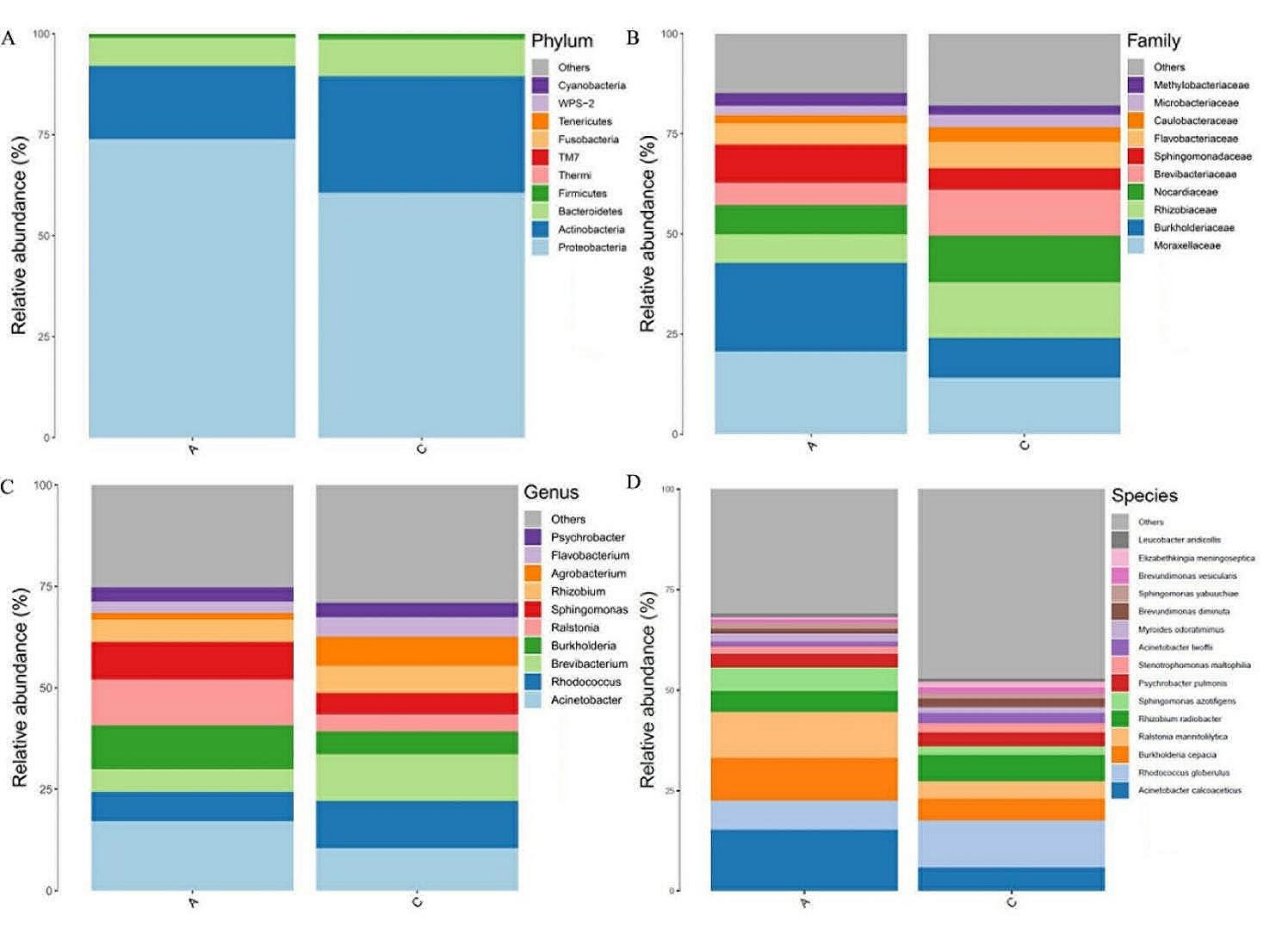
Relative abundance of top 10 microbial taxa at different classification levels in exosomes of patients with acute and chronic cholecystitis. **A** Phylum level. **B** Family level. **C** Genus level. **D** Species level

To further analyze the differential microbial communities in exosomes of patients with acute and chronic cholecystitis, we assessed the relative abundances of the top 10 taxa at the phylum, class, family, and species levels. At the phylum level, *Proteobacteria*, *Actinobacteria*, *Bacteroidetes*, and *Firmicutes* were the predominant phyla in both acute and chronic cholecystitis patients (Fig. 4A). Comparatively, in the Acute group, the relative abundance of *Actinobacteria* (*P* < 0.01), *Bacteroidetes* (*P* < 0.05), and *Firmicutes* (*P* < 0.05) significantly decreased, while *Proteobacteria* significantly increased (*P* < 0.01) compared to the Chronic group (Supplementary Table S2). At the family level, *Moraxellaceae*, *Burkholderiaceae*, *Sphingomonadaceae*, *Rhizobiaceae*, *Nocardiaceae*, and *Brevibacteriaceae* were the major families identified in both acute and chronic cholecystitis patients (Fig. 4B). Relative abundances of *Moraxellaceae* (*P* < 0.01), *Burkholderiaceae* (*P* < 0.01), and *Sphingomonadaceae* significantly increased (*P* < 0.01) in the Acute group compared to the Chronic group, while *Rhizobiaceae* (*P* < 0.01), *Nocardiaceae* (*P* < 0.01), and *Brevibacteriaceae* (*P* < 0.01) significantly decreased (*P* < 0.001, Supplementary Table S3). At the genus level, *Acinetobacter*, *Rhodococcus*, *Brevibacterium*, and *Burkholderia* were the predominant genera in both acute and chronic cholecystitis patients’ exosomes (Fig. 4C). Relative abundances of *Brevibacterium* (*P* < 0.001) and *Agrobacterium* (*P* < 0.01) significantly decreased in the Acute group compared to the Chronic group (Supplementary Table S4). At the species level, the most abundant microbial species in exosomes of both acute and chronic cholecystitis patients were *Acinetobacter calcoaceticus*, *Rhodococcus globerulus*, *Burkholderia cepacia*, *Ralstonia mannitolilytica*, *Rhizobium radiobacter*, and *Sphingomonas azotifigens* (Fig. 4D). In the Acute group, the relative abundances of *Acinetobacter calcoaceticus* (*P* < 0.01), *Burkholderia cepacia* (*P* < 0.01), and *Ralstonia mannitolilytica* (*P* < 0.001) significantly increased, while *Rhodococcus globerulus* (*P* < 0.001) significantly decreased compared to the Chronic group (Supplementary Table S5). These results further indicated significant differences in microbial communities between the Acute and Chronic groups.

### Analysis of the difference of the microflora in exosomes and prediction of biological function between the two groups

**Fig. 5:**
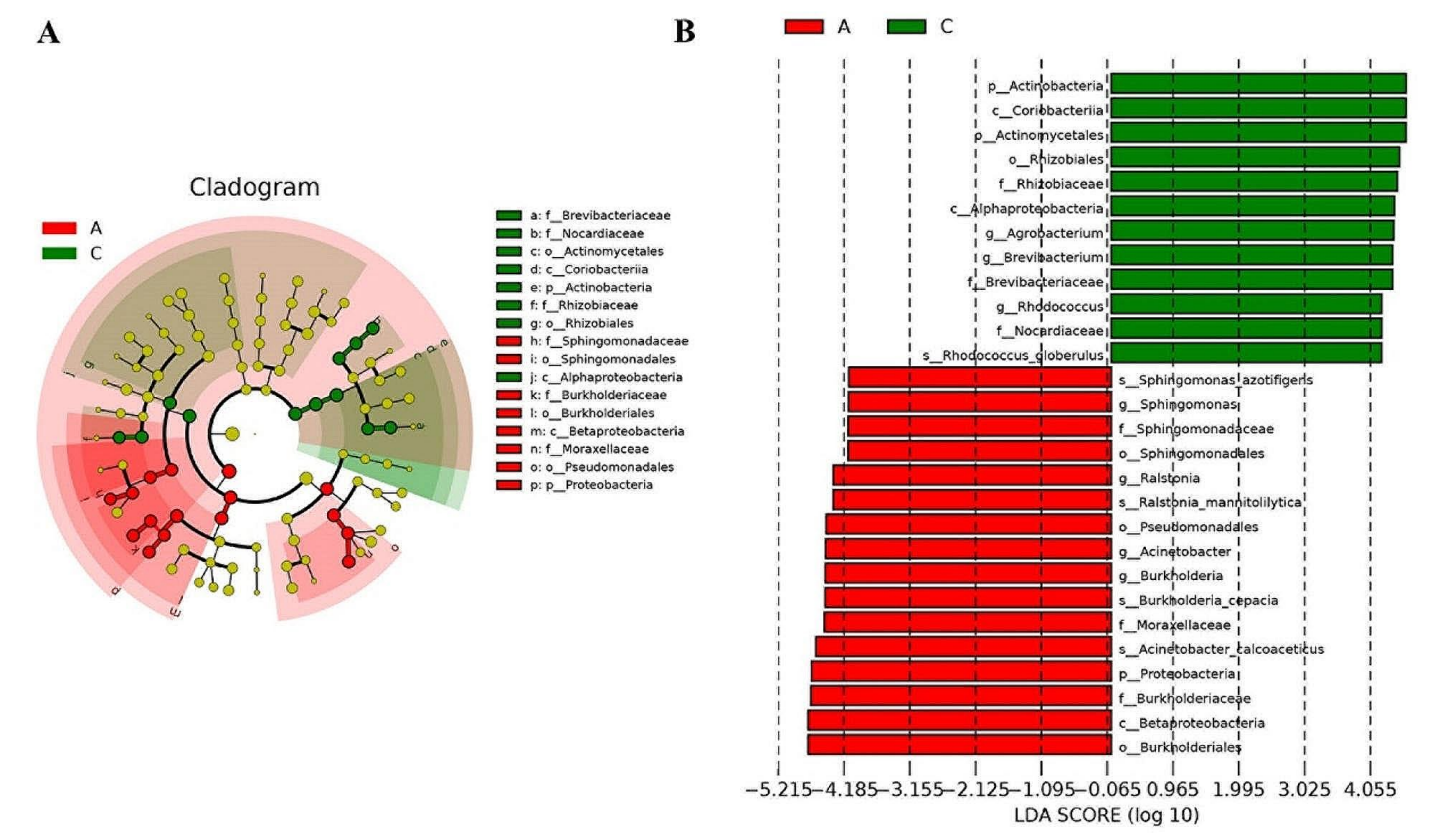
Differential composition of exosome microbiota in patients with acute and chronic cholecystitis. **A** Bacteria enriched in exosomes of the Acute group (red) and Chronic group (green). **B** Bar graph comparing the relative abundances of enriched taxa in exosomes of the Acute group (red) and Chronic group (green).

**Fig. 6:**
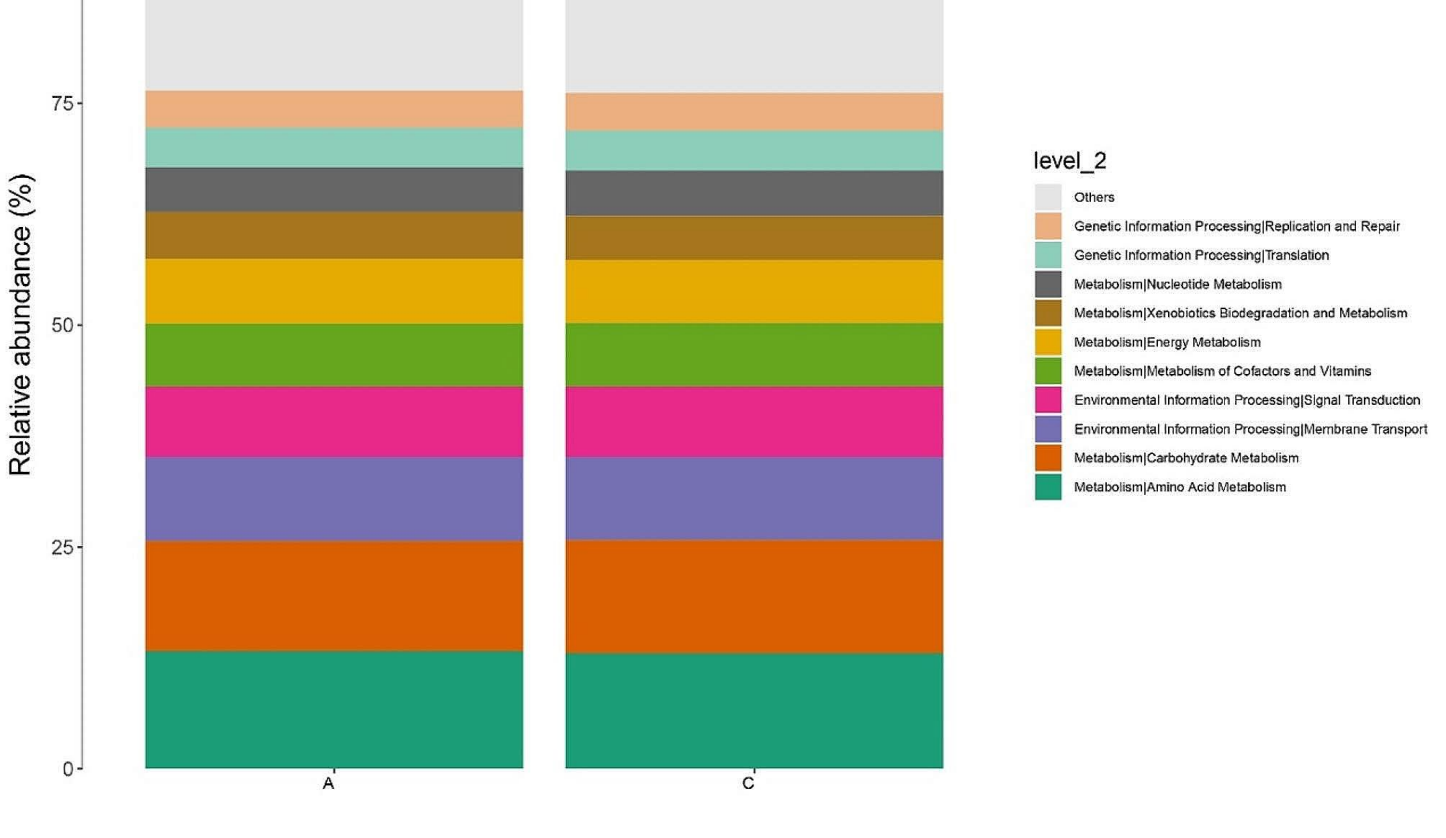
Tax4Fun functional prediction

Linear Discriminant Analysis Effect Size (LEfSe) based on logarithmic linear regression analysis (LDA score threshold set at ≥ 4) was employed to reveal key microbial taxa distinguishing the Acute and Chronic groups. Results indicated significant differences between the two groups at various taxonomic levels. At the phylum level, *Proteobacteria* exhibited a significant increase in relative abundance in the Acute group, while *Actinobacteria* were significantly elevated in the Chronic group. At the class level, *Betaproteobacteria* showed a significant increase in the Acute group, whereas *Alphaproteobacteria* and *Coriobacteriia* were significantly higher in the Chronic group. At the order level, *Burkholderiales*, *Pseudomonadales*, and *Sphingomonadales* were significantly elevated in the Acute group, whereas *Rhizobiales* and *Actinomycetales* were significantly higher in the Chronic group. At the family level, *Burkholderiaceae*, *Moraxellaceae*, and *Sphingomonadaceae* were significantly increased in the Acute group, whereas *Nocardiaceae*, *Brevibacteriaceae*, and *Rhizobiaceae* were significantly higher in the Chronic group. At the genus level, *Ralstonia*, *Acinetobacter*, *Burkholderia*, and *Sphingomonas* exhibited significant increases in the Acute group, while *Rhodococcus*, *Agrobacterium*, and *Brevibacterium* were significantly elevated in the Chronic group. At the species level, A*cinetobacter calcoaceticus*, *Ralstonia mannitolilytica*, *Burkholderia cepacia*, and *Sphingomonas azotifigens* showed significant increases in the Acute group, whereas *Rhodococcus globerulus* exhibited a significant increase in the Chronic group (Fig. 5A, B). All 28 identified microbial taxa demonstrated significant differences between the Acute and Chronic groups (*P* < 0.05). Furthermore, to predict the functional profiles of microbial communities in plasma exosomes from patients with acute and chronic cholecystitis, Tax4Fun analysis was performed, revealing differences in the enrichment levels of 36 pathways related to amino acid metabolism, carbohydrate metabolism, membrane transport, and signal transduction between the Acute and Chronic groups at the second level of KEGG pathways (Fig. 6).

## Discussion

Cholecystitis is an inflammatory disease of the digestive system, and exosomes are commonly studied as inflammatory mediators to investigate the pathogenesis of allergic diseases [33, 34]. Currently, most studies have focused on the bile and gut microbiota in healthy individuals and cholecystitis patients, with limited research on the microbial composition within exosomes of patients with acute and chronic cholecystitis. In this study, we analyzed the microbial communities in serum exosomes of patients with acute and chronic cholecystitis. Our findings revealed distinct microbial community characteristics in patients with acute cholecystitis as compared to those with chronic cholecystitis. Specifically, we observed reduced microbial diversity and specific changes in microbial abundance within the serum exosomes of patients with acute cholecystitis. Furthermore, diverse microbial metabolic pathways were also identified between the acute and chronic groups.

This study initially conducted α-diversity and β-diversity analyses on two groups of patients. Alpha-diversity serves as an indicator to measure species diversity within an ecosystem or community, while β-diversity metrics reveal relative differences in microbial communities between patients with acute and chronic cholecystitis [35]. Mora-Guzmán I et al. and Mintz D et al. found that there are differences in microbial profiles in the bile of cholecystitis patients compared to healthy individuals, with lower richness and diversity in patient microbiota [36, 37]. Similarly, in our study, we observed lower bacterial diversity and richness in the Acute group compared to the Chronic group, accompanied by significant alterations in microbial composition between the two groups. These findings indicated distinct microbial community compositions within exosomes in the blood of patients with acute and chronic cholecystitis.

Studies have shown the presence of diverse microbial communities in the biliary tract, where microbial dysbiosis might contribute to the formation of gallstones [17]. Previous research indicated that the *Firmicutes*, *Bacteroidetes*, *Actinobacteria*, and *Proteobacteria* dominate the bile bacterial composition [38–40]. In our study, we observed similar results, particularly noting a significant increase in *Proteobacteria* in the exosomes of patients with acute cholecystitis. *Proteobacteria* are highly abundant in the biliary tract [17], constituting approximately 1% of the healthy human gut microbiota [41]. However, in our study, *Proteobacteria* accounted for over 50% of both acute and chronic cholecystitis exosomes, suggesting an alteration in the microbial composition of cholecystitis patients. An investigation into bile microbiota identified Gram-negative bacteria such as *Escherichia coli*, *Enterobacteriaceae*, *Pseudomonas aeruginosa*, and *Klebsiella* as predominant pathogenic taxa in cholecystitis patients [42]. Notably, *Escherichia coli*, *Enterobacteriaceae*, *Pseudomonas aeruginosa*, and *Klebsiella* are members of the Proteobacteria phylum [43]. Among these, *Enterobacteriaceae* is a major harmful member of the human microbiota [44], associated with various diseases including acute pelvic inflammatory disease, necrotizing enterocolitis, and urinary tract infections [45–47]. Liu et al. [48] demonstrated that isolates of *Enterobacteriaceae* from patients with acute cholecystitis could induce inflammation and morphological changes in animal gallbladders. Additionally, *Enterobacteriaceae* can impair the intestinal barrier, leading to the translocation of intestinal microbiota to the host’s bloodstream and biliary system [45]. These findings indicate that bacteria from the *Enterobacteriaceae* family might be the primary pathogenic microorganisms in acute cholecystitis. Sarah J Powers et al. [49] detected *Pseudomonas aeruginosa* in gallbladder tissue samples from common marmosets (*Callithrix jacchus*) with cholecystitis. In a study on the microbiota of gallstones and bile, *Pseudomonas aeruginosa* exhibited the highest glucuronic acid enzyme activity and produced higher concentrations of phospholipase A2, promoting gallstone formation and, consequently, acute cholecystitis [50]. These findings suggest that *Pseudomonas aeruginosa* might serve as a diagnostic biomarker for acute cholecystitis. In our study, we observed significant differences in the microbial composition within exosome in the blood of patients with acute and chronic cholecystitis. Further analysis revealed significant disparities in the *Proteobacteria* phylum between the two patient groups. Combined with previous research results, we speculate that varying levels of *Proteobacteria* could potentially serve as novel biomarkers for diagnosing acute and chronic cholecystitis. Additionally, in our study, we observed a significant decrease in the abundance of *Firmicutes*, *Bacteroidetes*, and *Actinobacteria* phyla in the exosome of patients with acute cholecystitis, indicating potential alterations in these microbial communities in both acute and chronic cholecystitis patients. However, the specific mechanisms underlying these changes require further in-depth investigation.

The microbial community can influence various host metabolic reactions, disease progression, and signaling pathways, thereby regulating growth processes and the onset of chronic diseases [24, 51]. In this study, functional metabolism and physiological pathways affected by acute and chronic cholecystitis were predicted through Tax4Fun analysis. Our findings revealed differential enrichment in 36 pathways between the two patient groups, including amino acid metabolism, carbohydrate metabolism, membrane transport, and signal transduction. Amino acid metabolism encompasses several amino acids. For instance, histidine, as an anti-inflammatory amino acid, can reduce the levels of reactive oxygen species in the body. Studies have shown that histidine expression is decreased in patients with chronic cholecystitis, thereby promoting inflammatory responses in cholecystitis [52]. Moreover, decreased histidine concentrations have been observed in inflammatory chronic kidney diseases [53]. Our study further suggested that the microbial composition in patients with acute and chronic cholecystitis might impact these metabolic pathways.

Currently, the diagnosis of cholecystitis primarily relies on imaging examinations such as ultrasound, CT, and HIDA scans, supplemented by comprehensive evaluation of detailed medical history, thorough clinical examinations, and laboratory test results [54]. These diagnostic modalities have been demonstrated to possess good accuracy in clinical practice. This study revealed significant differences in the microbial communities within serum exosomes of patients with acute and chronic cholecystitis. However, due to the time-consuming nature of microbial analysis and the imperative for immediate surgical intervention once acute cholecystitis is diagnosed, the clinical application of microbial analysis remains supplementary. Gallstones are considered one of the predisposing factors for biliary tract infections. Meanwhile, there have been significant changes in the distribution and resistance of pathogenic microorganisms causing biliary tract infections [55]. In the early stages of acute cholecystitis, bile is typically sterile and becomes infected as a secondary event [56]. Previous studies have indicated that approximately 9-42% of patients undergoing elective laparoscopic cholecystectomy develop bile infections, with the incidence of positive bile cultures in patients with acute cholecystitis rising to 35–65% [56], and to 44% in patients with chronic cholecystitis [55]. For patients with moderate to severe acute cholecystitis, early pathogen eradication based on antibiotics is crucial in limiting systemic sepsis and local inflammation following cholecystectomy [57]. Appropriate initial antibiotic therapy should not be delayed while waiting for culture test results, as this delay may increase the mortality rate of patients with biliary tract infections [43]. Hence, appropriate antibiotic therapy should commence immediately following diagnosis [38]. Gram-negative bacterial strains constitute the major pathogenic microbial population in cholecystitis, with typical gram-negative bacteria (such as *Escherichia coli*, *Pseudomonas aeruginosa*, and *Klebsiella pneumoniae*) belonging to the *Proteobacteria* family [42]. In this study, we found that *Proteobacteria* accounted for over 50% of the exosomal microbiota in patients with both acute and chronic cholecystitis. It has been shown that Gram-negative bacterial strains have low susceptibility to cephalosporins, quinolones, and ampicillin [42]. Therefore, alternative antibiotics may need to be considered when selecting antibiotic therapy. These findings suggest that microbial analysis could provide better guidance for treatment [42].

However, this study has certain limitations. Firstly, we only observed changes in the microbial composition of serum exosomes in patients with acute and chronic cholecystitis in this study. However, existing research suggests that the gut microbiota might also be associated with gallbladder diseases. Secondly, the composition of microbiota is influenced by various factors, but this study did not assess the impact of other factors on microbial changes, such as age, gender, diet, and lifestyle. Additionally, once clinically diagnosed with acute cholecystitis, patients require early surgical treatment, while microbial composition analysis may require more time. Therefore, there is a need for further optimization of the workflow for microbiota analysis in the future. Despite these limitations, the findings of this study offer a novel insight into the role of microbial composition in acute and chronic cholecystitis, providing a new approach to the diagnosis of these conditions. However, the conclusions of this study are currently applicable only at the laboratory level. Future research should be conducted on a larger scale to explore the role of microbiota in acute and chronic cholecystitis and to optimize the technology for wider clinical application in the future.

## Conclusion

In summary, our study demonstrated significant differences in the composition and diversity of microbiota in serum exosomes between patients with acute and chronic cholecystitis. The notable differences in the abundance of *Proteobacteria*, *Actinobacteria*, *Bacteroidetes*, and *Firmicutes* hold the promise of serving as novel biomarkers for the clinical diagnosis of acute and chronic cholecystitis. This research contributes to the diagnosis of clinical acute and chronic cholecystitis and provides a theoretical foundation for elucidating the potential mechanisms of microbial metabolism.

### Electronic supplementary material

Below is the link to the electronic supplementary material.

Supplementary Material 1

Supplementary Material 2

Supplementary Material 3

Supplementary Material 4

Supplementary Material 5

## Data Availability

The sequencing raw data have been deposited into the Sequence Read Archive Database of National Center for Biotechnology Information (NCBI). The BioProject accession number is PRJNA1049960 (https://www.ncbi.nlm.nih.gov/sra/?term=PRJNA1049960).
